# Postharvest Preservation of Red Apples Using Edible Coatings and Packaging

**DOI:** 10.1002/fsn3.71538

**Published:** 2026-02-20

**Authors:** Mohsen Azadbakht, Mohammad Vahedi Torshizi, Alireza Sabaghi Khatunabadi, Farimah karimzadeh viyarsagh, Feryal Varasteh Akbarpour

**Affiliations:** ^1^ Department of Biosystem Engineering Gorgan University of Agricultural Sciences and Natural Resources Gorgan Iran; ^2^ Biosystems Engineering Department Tarbiat Modares University Tehran Islamic Republic of Iran; ^3^ Department of Horticulture Gorgan University of Agricultural Sciences and Natural Resources Gorgan Iran

**Keywords:** edible coating, packaging methods, postharvest quality, Red Delicious apple, spermidine

## Abstract

Limited comparative evidence exists on how polyamine‐based edible coatings, in combination with various packaging systems, simultaneously influence the physical stability and biochemical quality of “Red Delicious” apples during storage. In this study, the effects of edible coatings (spermidine, putrescine, and chitosan) and packaging methods (unpacked, plastic film, and zipper bags) on physical properties (length, thickness, geometric mean diameter, weight, and surface area) and chemical attributes (pH, total soluble solids, total phenolics, flavonoids, and antioxidant activity) were evaluated. ANOVA results indicated that both coatings and packaging significantly influenced apple quality during storage, whereas their interaction effects were generally negligible. Packaging played a more critical role in maintaining dimensional stability, while coatings—particularly spermidine—were more effective at reducing weight loss and geometric alterations. Conversely, coatings significantly preserved phenolic compounds and antioxidant activity, whereas packaging exerted a stronger influence on pH and total soluble solids. Among the packaging treatments, zipper bags demonstrated the best performance in minimizing moisture loss and preserving dimensional integrity. Spermidine coating, on the other hand, showed the greatest efficacy in maintaining both chemical and physical quality attributes. Overall, the combination of spermidine coating and zipper bag packaging is recommended as an efficient and cost‐effective postharvest strategy. This approach not only reduces postharvest losses and extends shelf life but also preserves the nutritional and market value of apples, offering substantial economic and export benefits for the horticultural industry. These findings provide actionable guidance for postharvest management and highlight the potential of integrated coating–packaging interventions to reduce losses and enhance value in fruit supply chains.

## Graphical abstract

Spermidine coating combined with zipper bag packaging significantly reduced weight loss and preserved phenolic content in “Red Delicious” apples during cold storage. This integrated approach maintained dimensional stability, antioxidant activity, and marketable quality for over 30 days. It offers a cost‐effective, eco‐friendly postharvest strategy to extend shelf life and support export‐oriented horticultural supply chains.

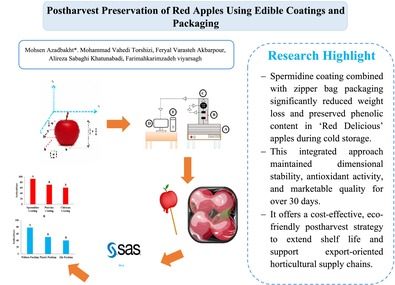

## 1 Introduction

Fruits typically soften during ripening—whether on the tree or in storage—due to a reduction in cell wall firmness (Azadbakht et al. 2018). These physical and biochemical changes in harvested fruits, often exacerbated by various postharvest stresses, lead to deterioration in quality and substantial agricultural losses. Numerous factors during storage act as stressors that compromise fruit quality and shorten shelf life (Jafarzadeh et al. 2023).

Unlike engineered materials, agricultural products possess unique qualitative traits that are frequently quantified to facilitate handling, grading, and postharvest management. Fruit appearance significantly influences market value, underscoring the importance of proper postharvest practices. Enhancing physical attributes not only improves marketability but also supports export potential (Azadbakht, Torshizi, et al. 2022).

Researchers have long focused on the relationships among key physical properties of fruits, such as mass, volume, and dimensions. These parameters determine packaging efficiency—that is, the number of fruits that can be accommodated in a given container—and help predict optimal drying rates, thereby reducing energy consumption and processing time. Among these attributes, mass, volume, and surface area are particularly critical for sizing and grading systems (Jafarzadeh et al. 2023; Mahmoodi and Azadbakht 2021).

The shape of horticultural products is a key determinant of heat transfer—both within plant tissues and between the produce and its surrounding environment. Accurate geometric models are therefore essential when employing numerical simulations to analyze physiological processes such as cooling (Dehghannya et al. 2010).

A thorough understanding of the physical, chemical, and mechanical properties of agricultural products is critical for growers, engineers, machinery designers, food scientists, processors, and consumers alike. During harvest, fruits are often exposed to mechanical stresses—such as impact from falling to the ground or colliding with tree trunks—which can cause bruising and lead to significant economic losses. Knowledge of these properties enables optimized handling practices throughout the postharvest chain, from harvest through transportation, packaging, and storage (Azadbakht et al. 2024).

Specific physical characteristics, including surface area and projected area, play a pivotal role in modeling water loss, gas exchange, heat transfer, pesticide deposition, respiration rate, growth dynamics, quality assessment, ripening indices, and the prediction of optimal harvest timing and packaging requirements (Azadbakht, Jafarzadeh, et al. 2022; Azadbakht et al. 2024; Azadbakht, Mahmoodi, and Vahedi Torshizi 2019).

Long‐term storage and packaging of fruits—particularly seasonal varieties—have historically been essential for maintaining quality and ensuring year‐round availability. In recent decades, food safety and hygiene have emerged as major global priorities. Ensuring the safety and quality of packaged fruits and fruit products is now a critical challenge in international food supply chains (Kalia and Parshad 2015).

The food industry has developed a wide array of packaging materials and formats to address these needs. These materials—such as glass, plastic, metal, and cardboard—are selected based on the specific characteristics of the produce, as their functional properties (e.g., permeability, mechanical strength, and barrier performance) directly influence shelf life and product integrity (Opara et al. 2015).

Optimizing storage conditions is critical for maintaining kiwifruit quality. Any intervention that minimizes water loss and slows metabolic activity can effectively extend shelf life and preserve quality.

Previous studies have demonstrated that a wide range of fruits—including apples, pears, citrus varieties (oranges, tangerines, grapefruits, lemons), melons, lychees, cucumbers, pineapples, bananas, mangoes, and avocados—benefit from edible coatings. These coatings mitigate water loss, suppress physiological disorders, prevent surface dulling, reduce disease incidence, and ultimately prolong storage life (Janick 2003).

Edible coatings function by modulating respiration rate, inhibiting microbial growth, and—most critically—reducing moisture loss. They are typically composed of non‐toxic, biodegradable, and edible materials (e.g., waxes or polysaccharides) that pose no risk to human health or the environment, effectively suppress fungal development, and help maintain the fruit's visual appeal over extended periods (Ardakani et al. 2010).

Polyamines have been shown to delay ripening and senescence—the final stage of fruit development and an integral part of the fruit's life cycle (Gao et al. 2021). Post‐ripening oxidative processes degrade cell wall components, leading to tissue softening (Sood and Nagar 2008). Polyamines are low‐molecular‐weight organic polycations present in nearly all living organisms and are involved in diverse physiological processes, including growth, development, and stress tolerance (Valero et al. 2002). In plants, the primary polyamines—putrescine, spermidine, and spermine—interact with anionic macromolecules such as DNA, RNA, phospholipids, and specific proteins due to their polycationic nature, thereby stabilizing cellular structures and enhancing postharvest resilience (Heby and Persson 1990).

Although previous studies have investigated the effects of polyamine‐based coatings or packaging on fruits such as persimmon, banana, mango, and plum, limited comparative evidence exists for apples—particularly regarding the combined effects of spermidine coating and different packaging systems. No study has simultaneously evaluated how spermidine interacts with packaging atmosphere to influence both physical stability and biochemical quality during storage. This gap is significant for Red Delicious apples, which are highly susceptible to shrinkage and quality loss during long‐term storage.

The primary objective of this study was to evaluate the effects of three edible coatings—spermidine, putrescine, and chitosan—and three packaging methods (unpacked, plastic film, and zipper bag) on the physical and chemical properties of “Red Delicious” apples during storage. The research aimed to identify the most effective coating–packaging combination for extending shelf life, minimizing postharvest losses, and preserving both the nutritional quality and marketability of the fruit.

## 2 Materials and Methods

### 2.1 Sample Preparation

Apples (*Red Delicious*) were obtained from a reputable commercial supplier in Golestan Province, Iran. To minimize variability due to fruit maturity, apples were selected based on the commercial maturity indices commonly used in postharvest studies of the Red Delicious cultivar. The fruits were uniform in size, shape, and external color, and were free of mechanical damage or disease. Maturity was determined using visual and physical indicators typical of the commercial harvest stage, including 80%–90% red surface coverage, total soluble solids (TSS) of about 12°–13° Brix, initial titratable acidity and pH values recorded at the start of the experiment, and a moisture content of 32% measured according to AOAC standards (Azadbakht, Jafarzadeh, et al. 2022). These combined criteria ensured that all apples were at a similar commercial maturity stage and suitable for storage experiments, and only fruits with uniform appearance and without bruising or visible defects were selected.

### 2.2 Determination of Physical Properties

**FIGURE 1:**
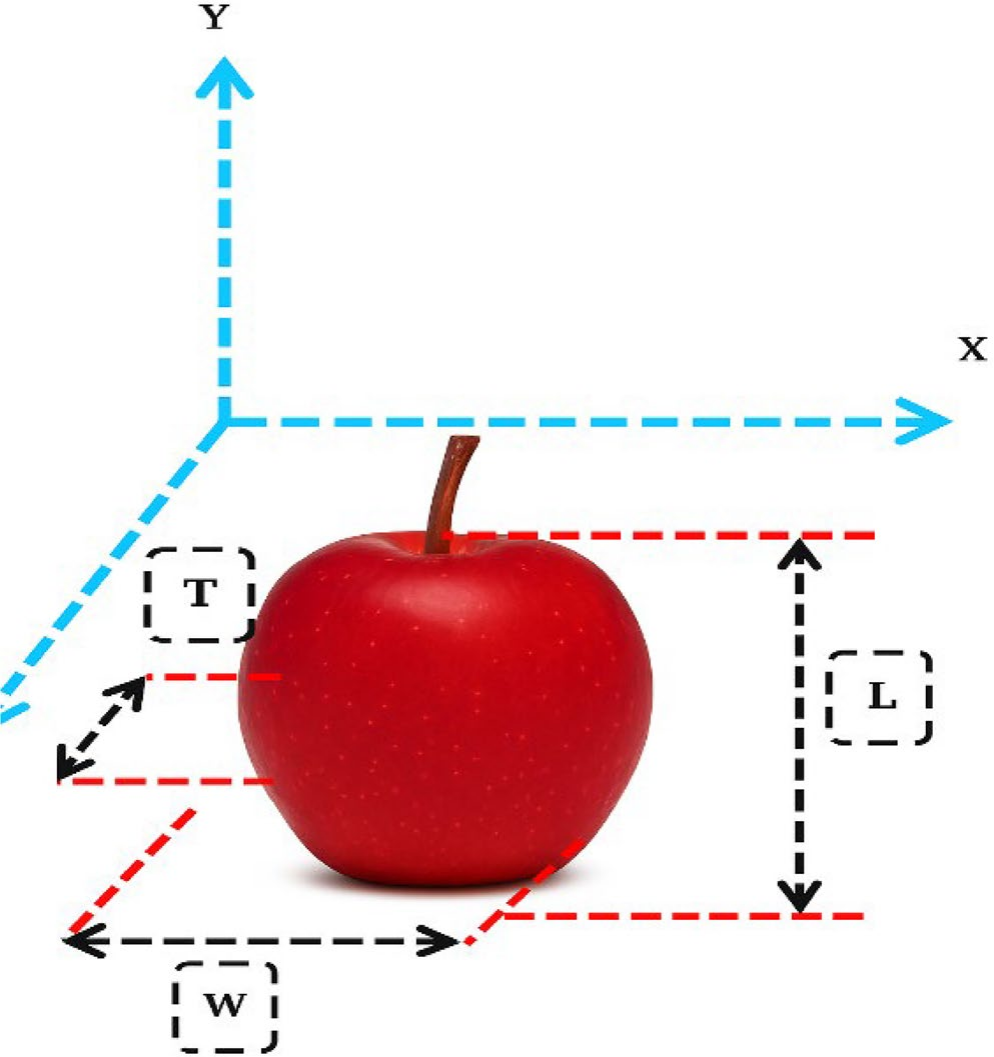
*L*: Length, *W*: Width, and *T*: Thickness are considered for the red apple.

To determine the physical properties of “Red Delicious” apples prior to storage, representative samples were weighed using a digital balance with an accuracy of 0.001 g. The average dimensions were measured using a digital caliper (±0.05 mm) to record the longitudinal diameter (*L*), transverse diameter (*W*), and thickness (*T*). Figure 1 illustrates the dimensional parameters used for geometric characterization of the apples.

Accordingly, the equivalent diameter ($D_{p}$), geometric mean diameter ($D_{g}$), and sphericity (ϕ) were computed using the equations provided in Table 1.

**TABLE 1:** Equations for equivalent diameter ($D_{p}$), geometric mean diameter ($D_{g}$), sphericity (ϕ), surface area (*S*), and elongation ratio (*R*) for the apple.

Formula	References
$D_{p}={\left(\frac{L{\left(W+T\right)}^{2}}{4}\right)}^{\frac{1}{3}}$	Azadbakht et al. (2018)
$D_{g}$ = (*LWT*)$\frac{1}{3}$	Jafarzadeh et al. (2023)
$\phi =\frac{{\left(\mathrm{LWT}\right)}^{\frac{1}{3}}\;}{L}$ (3)	Azadbakht, Vahedi Torshizi, and Mahmoodi (2019)
$S=\frac{\mathrm{\pi B}L^{2}}{2L-B}$ (4)	Kheiralipour et al. (2008)
$B={\left(\mathrm{WT}\right)}^{0.5}$ (5)	Li et al. (2010)
$R=\left(\frac{W}{L}\right)\times 100$	Karababa (2006)

### 2.3 Quasi‐Static Test

**FIGURE 2:**
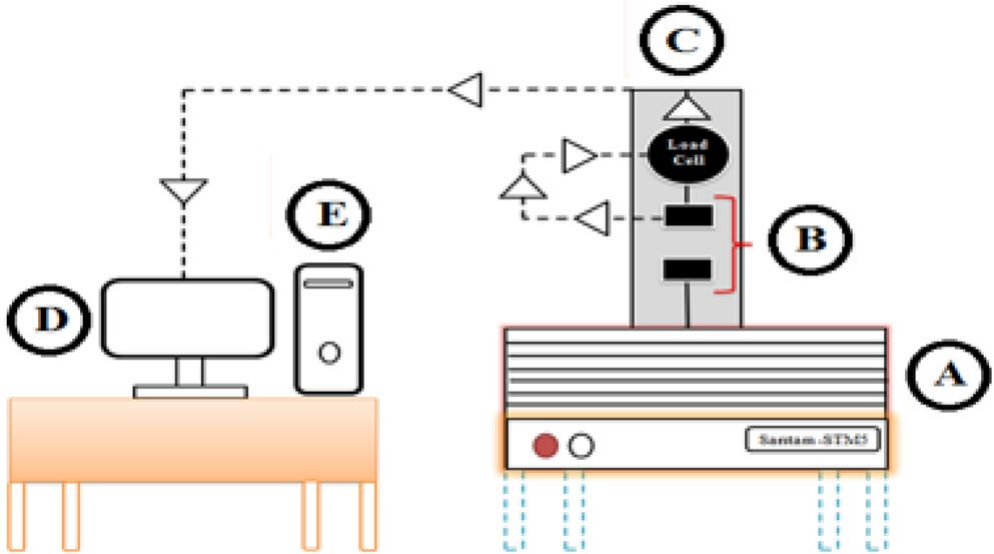
Schematic diagram of quasi‐static loading of the red apple. (A) Instron machine; (B) flat‐edge plate; (C) load cell; (D) computer; (E) output data.

For the mechanical compression test under flat‐edge loading, a universal testing machine (Instron Santam STM‐5) equipped with a 500 N load cell was used. The test was conducted using two parallel circular plates at a crosshead speed of 5 mm/min, applying three force levels (50, 70, and 100 N), with three replicates per force level (Figure 2). Individual “Red Delicious” apples were positioned horizontally between the plates and subjected to uniaxial compression, while the deformation response and time were recorded throughout the process.

**FIGURE 3:**
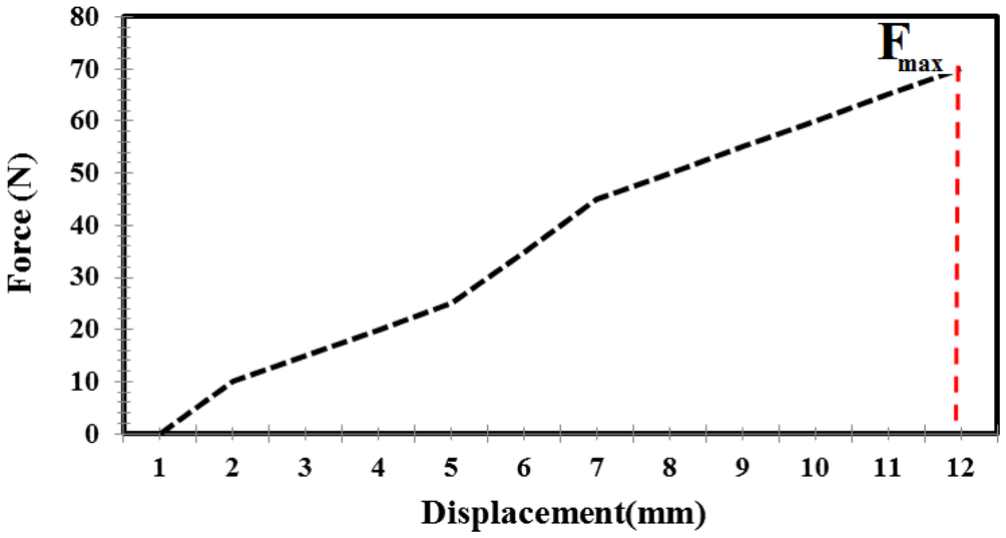
Force–deformation curve for red apple.

The movable plate continued to compress the fruit until the target force was reached, as monitored by the computer. The applied load was then stopped, and the force–displacement curve was plotted (Figure 3) (Azadbakht and Vahedi Torshizi 2020).

### 2.4 Measurement of the Chemical Properties of the Apple Fruit

All physical and chemical analyses were carried out on different samples and in three independent replications. The chemical properties of the fruit samples—including total phenolics, flavonoids, antioxidant activity, total soluble solids (TSS), total sugars, titratable acidity (TA), moisture content, and pH—were analyzed at the beginning (as control) and at the end of the storage period for each treatment group. All analyses were conducted in the Laboratory of Horticulture, Gorgan University of Agricultural Sciences and Natural Resources. The specific methodologies, protocols, and equipment used are detailed below. All chemical analyses described in Sections 2.4.1, 2.4.6 were performed with three independent replicates per treatment, and each replicate consisted of a single sample extracted separately and prepared according to the methanolic extraction protocol described below.

Fresh fruit tissue (5 g) was homogenized with 20 mL of 80% (v/v) aqueous methanol and incubated for 30 min at 25°C (room temperature) under continuous shaking. The mixture was then centrifuged at 6000 rpm for 10 min, and the supernatant was filtered through Whatman No. 1 filter paper. The resulting extract was stored at 4°C in the dark and used for subsequent chemical analyses on the same day.

#### 2.4.1 Total Phenol

Exactly 1 g of apple pulp was homogenized with 10 mL of 80% methanol using a laboratory homogenizer for 2 min. The mixture was incubated in a shaker at 25°C for 60 min to allow complete extraction. The extract was centrifuged at 4000 rpm for 10 min, and the supernatant was collected. The supernatant was filtered through Whatman No. 1 filter paper to obtain a clear methanolic extract. This extract was used immediately for the determination of phenolic content.

The Folin–Ciocalteu method was used to determine total phenolic content. The Folin–Ciocalteu reagent was freshly diluted 1:10 (v/v) with distilled water prior to use. Then, 20 μL of methanolic extract was mixed with 100 μL of the diluted Folin–Ciocalteu reagent and 1.16 mL of distilled water. After 5 min of rest, 300 μL of 1 M sodium carbonate solution (10.6 g per 100 mL distilled water) was added. The mixture was incubated in a steam bath at 40°C for 30 min. Absorbance was recorded at 765 nm using a Camspec M501 spectrophotometer. Phenolic content was calculated using equation (1) and expressed as milligrams of gallic acid per gram of sample (Jaramillo‐Flores et al. 2003).

(1) y=0.5x−0.005

#### 2.4.2 Total Flavonoids

To measure the flavonoid content of the fruit, 0.5 mL of methanolic extract along with 1.5 mL of methanol, 0.1 mL of 10% (w/v) aluminum chloride in ethanol (10 g in 100 mL), 0.1 mL of 1 M potassium acetate (2.41 g in 10 mL distilled water), and 2.8 mL of distilled water were mixed. Instead of the methanolic extract, only pure methanol was used to prepare the control. The mixture was placed in the dark for 30 min and immediately scanned by a spectrophotometer at 415 nm; the device's output was recorded. The obtained flavonoid numbers were corrected using the standard curve. The standard curve was obtained using the formula (0–1). For this purpose, different concentrations of the quercetin standard were prepared, and after determining the absorbance (*y*), the actual concentrations and the total flavonoid concentration (*x*) were obtained.

(2) y=0.036x+0.0996

#### 2.4.3 Percentage of Inhibition of Free Radicals by the DPPH Method

In this experiment, the inhibition percentage of DPPH free radicals was measured using the method described by Bondet et al. (1997). First, 2 mL of 0.1 mM DPPH solution (4 mg DPPH dissolved in 100 mL methanol) was added to the test tube, followed by 2 mL of the prepared methanolic extract. Then, the test tubes were placed in a dark environment for 30 min, and the absorbance was immediately measured at 517 nm on a spectrophotometer. The control sample consisted of 2 mL of DPPH and 2 mL of methanol. The spectrophotometer was calibrated with methanol. The numbers obtained by formula three were converted to antioxidant activity (Li et al. 2012).

(3) $$\text{DPPH}=\frac{A_{c}-A_{s}}{A_{c}}\times 100$$

where *A*_c_, absorbance of the control sample; *A*_s_, absorption of samples.

#### 2.4.4 Measurement of Dissolved Solids

In the laboratory, fruit extract was extracted. Then, using a filter paper, a few drops of the extract were poured onto the refractometer, allowing the amount of dissolved solids to be calculated. Also, the digital refractometer (MT‐032ATC) was made in Taiwan (Seyedabadi et al. 2017).

#### 2.4.5 Titratable Acidity Measurement

Titratable acidity was determined by titration with 0.1 N sodium hydroxide (NaOH). Briefly, 2 mL of apple juice was diluted with 38 mL of distilled water, and three drops of 1% (w/v) phenolphthalein in ethanol were added. The mixture was titrated with 0.1 N NaOH until a faint pink endpoint persisted for 30 s (pH ≈8.2).

Results were expressed as malic acid (%), calculated using the following equation:

$$\mathrm{TA}\;\left(\%\text{malic acid}\right)=\frac{V\times N\times 0.067\times 100}{W}$$

where V = volume of NaOH used (mL), N = normality of NaOH, 0.067 = equivalent weight of malic acid (g/eq), W = volume of sample (mL).

The ratio of total soluble solids to titratable acidity (TSS/TA) was calculated as a taste index (Patel et al. 2019).

#### 2.4.6 pH Value

The pH of apple juice extract was measured directly using a calibrated digital pH meter (model AD100, Hungary; ±0.01 accuracy). Before measurement, the instrument was calibrated with standard buffer solutions at pH 4.00 and pH 7.00. For each sample, 10 mL of the fruit extract was placed in a beaker, and the electrode was immersed until a stable reading was obtained. pH values were recorded in three independent replicates for each treatment (Valentines et al. 2005; Martín‐Diana et al. 2009).

### 2.5 Preparation of Coating Solutions

Spermidine and putrescine coating solutions were prepared at a concentration of 1 mM each, following standard protocols reported in postharvest studies on apple and other fruits (Azadbakht, Jafarzadeh, et al. 2022). Aqueous solutions were freshly prepared and used within 2 h of preparation.

### 2.6 Statistical Analysis

Samples subjected to quasi‐static loading were stored for 20 days before imaging. All experiments were conducted in triplicate, and data were analyzed using a factorial design in SAS. Correlation analysis was performed to evaluate the relationships between geometric diameters, equivalent diameter, surface area, elongation ratio, sphericity, and chemical properties.

### 2.7 Principal Component Analysis (PCA)

In this study, collected data were analyzed using The Unscrambler X 10.4 (64‐bit). Initially developed by Harald Martens and later expanded by CAMO, this software is a specialized tool for analyzing multivariate data. Key features include data calibration, predictive modeling, and advanced chemometric methods. PCA was employed to examine correlations between different treatments (packaging and coating; scores) and physical properties (loadings). By transforming correlated variables into a smaller set of independent principal components, PCA reduces data dimensionality, eliminates noise, and retains the maximum variance. This not only compressed the dataset but also allowed for a more straightforward interpretation of the underlying structure. As an unsupervised method, PCA does not require a dependent variable; instead, it identifies intrinsic relationships within the data. This makes it highly effective for exploratory studies and pattern recognition. Ultimately, PCA enabled a more accurate evaluation of variable relationships and the development of more reliable models (Kamboj et al. 2020).

## 3 Result and Discussion

### 3.1 Statistical Analysis Result

The ANOVA (Table 2) results revealed that both coating and packaging had significant but largely independent effects on the physical attributes of apples, with the interaction being largely nonsignificant. Packaging played a dominant role in maintaining dimensional stability, including length, height, thickness, and equivalent diameter. In contrast, coating was more effective in reducing weight loss and influencing specific geometric indices, such as geometric diameter. These findings indicate that packaging is crucial for preserving fruit dimensions during storage, whereas coatings primarily control moisture loss and shape‐related parameters. Since interactions were generally absent, coating and packaging strategies can be optimized independently; however, the near‐significant interaction observed for length highlights the need for further analysis to better understand the potential combined effects.

**TABLE 2:** Analysis of variance results for the effects of coating and packaging on physical properties and bruising percentage of apples.

Variables	DF	Percentage of length changes		Percentage of height changes	
		Mean square	*F*	Mean square	*F*
Coating	2	41.76	42.47**	82.15	19.14**
Packaging	2	84.409	85.82**	100.09	23.32**
Coating × Packaging	4	2.47	2.51ns	2.109	0.49ns
		Percentage of thickness changes		Percentage of weight change	
Coating	2	58.33	34.53**	65.98	84.95**
Packaging	2	95.80	56.72**	42.35	54.53**
Coating × Packaging	4	1.50	0.89ns	0.64	0.83ns
		Geometric diameter		Equivalent diameter	
Coating	2	0.0645	7.76**	60.57	36.00**
Packaging	2	0.0049	0.59ns	93.57	55.61**
Coating × Packaging	4	0.0190	2.29ns	1.35	0.81

*Note:* ** Significant at level 1%; ns, insignificant.

#### 3.1.1 The Length of an Apple Fruit

**FIGURE 4:**
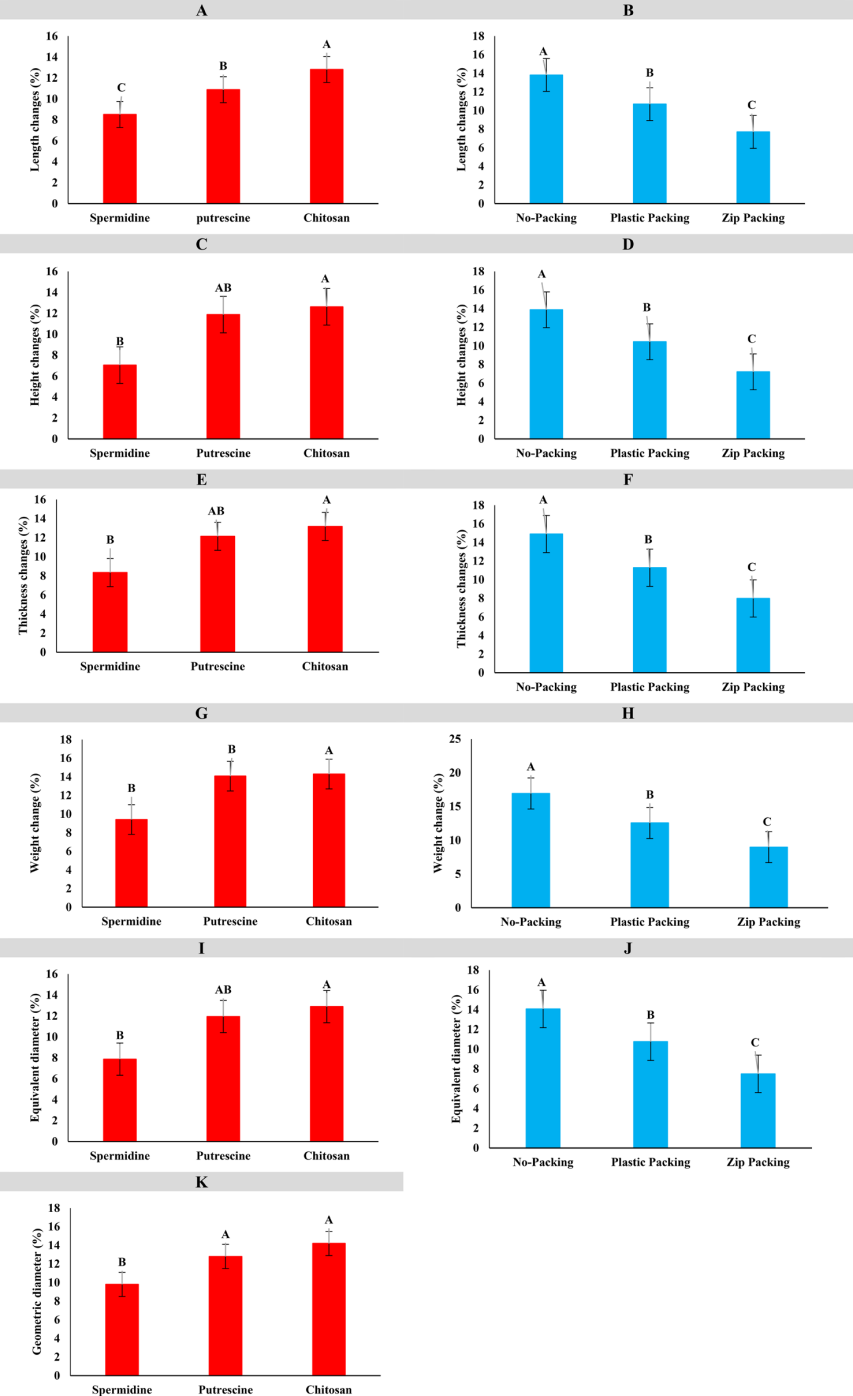
Comparison of the mean physical changes in the apple fruit. In each chart, similar letters indicate no significant difference, while different letters indicate a significant difference at each level.

In Figure 4A, the effects of three coating treatments—spermidine, putrescine, and chitosan—on apple length changes are shown. Chitosan induced the highest length change (~13%, group A), likely due to its permeability affecting internal tissue moisture and turgor, whereas spermidine minimized changes (~8%, group C) by forming a protective layer that preserved structural stability; putrescine showed intermediate effects (~11%, group B). Figure 4B presents the influence of packaging: apples with no packaging exhibited the most tremendous length change (~14%, group A), plastic film reduced it to ~11% (group B), and zipper packaging was most effective (< 9%, group C), likely by stabilizing moisture and limiting gas exchange.

Coating and packaging treatments significantly influenced the physical quality of apples during storage. Among coatings, chitosan induced the highest changes in fruit height, thickness, weight, and both geometric and equivalent diameters (~12%–14%), followed by putrescine (~12%–13%), while spermidine consistently minimized these changes (~7%–9%). Similarly, packaging had a substantial effect, with no packaging leading to the greatest dimensional and weight changes (~14%–15%), plastic film reducing changes (~11%–12%), and zipper packaging being the most effective (~8%–10%). Overall, spermidine coating combined with zipper packaging provided the most stable physical characteristics across all measured parameters, demonstrating its potential to preserve postharvest apple quality.

The superior ability of spermidine to maintain physical stability is likely due to its protective effects at the cellular level. Spermidine stabilizes cell membranes, reduces respiration rates, and limits moisture loss, thereby minimizing tissue shrinkage and dimensional alterations. In contrast, chitosan and putrescine may facilitate greater gas and water exchange, leading to greater physical changes. This mechanistic understanding emphasizes that spermidine's effectiveness is not only reflected in numerical measurements but also in its ability to maintain tissue integrity and cellular function during storage.

Although chitosan‐treated apples showed the highest TSS and pH values, spermidine‐treated fruits exhibited superior phenolic content and antioxidant activity. This discrepancy occurs because TSS and pH primarily reflect sugar and acid content, whereas antioxidant levels are influenced by phenolic metabolism and the protection of cellular structures. Spermidine's membrane‐stabilizing and respiration‐limiting effects may preserve or enhance phenolic compounds, thereby increasing antioxidant capacity independently of sugar accumulation. Thus, the biochemical advantages of spermidine can coexist with lower TSS and pH, reconciling the apparent differences observed between physical–chemical and antioxidant trends.

### 3.2 Chemical Section

Table 3 summarizes the ANOVA results for the effects of coating, packaging, and their interaction on apple quality parameters, including pH, total soluble solids (TSS), phenolic compounds, flavonoids, and antioxidant activity. The data indicate that coatings significantly affected pH, TSS, phenolic content, and antioxidant activity, suggesting that coating materials can help regulate acidity, promote the accumulation of soluble solids, and preserve antioxidant properties. In contrast, their effect on flavonoids was not significant. Packaging exerted a more pronounced influence, significantly affecting pH, TSS, phenolics, and antioxidant activity, highlighting its critical role in maintaining physicochemical stability by controlling gas and moisture exchange; only flavonoids remained largely unaffected by packaging, likely due to their inherent stability. Notably, the interaction between coating and packaging was nonsignificant for all measured parameters, indicating that each factor acts essentially independently, with no apparent synergistic or antagonistic effects. Overall, these findings highlight that while coatings are crucial for enhancing antioxidant retention and regulating acidity, packaging plays a pivotal role in preserving apples' overall quality. Combining effective coatings with suitable packaging provides an optimal approach to postharvest management.

**TABLE 3:** Analysis of variance (ANOVA) results for the effects of coating and packaging on the chemical properties of apple fruit.

Variables	DF	pH		TSS	
		Mean square	*F*	Mean square	*F*
Coating	2	0.111	8.85**	10.67	6.52**
Packaging	2	0.285	22.71**	36.45	22.25**
Coating × Packaging	4	0.019	1.53ns	2.16	1.32ns
		Phenol (mg GAE/g FW)		Flavonoid (mg QE/g FW)	
Coating	2	7530.74	2.15*	0.0001	0.26ns
Packaging	2	14,108.25	4.60*	0.0017	2.50ns
Coating × Packaging	4	698.90	0.23ns	0.0003	0.45ns
		Antioxidant (%)			
Coating	2	21,374.10	9.64**		
Packaging	2	9478.59	4.57*		
Coating × Packaging	4	1444.04	0.65		

*Note:* ** Significant at level 1%; * Significant at 5% level; ns, insignificant.

#### 3.2.1 pH of Apple Fruit

**FIGURE 5:**
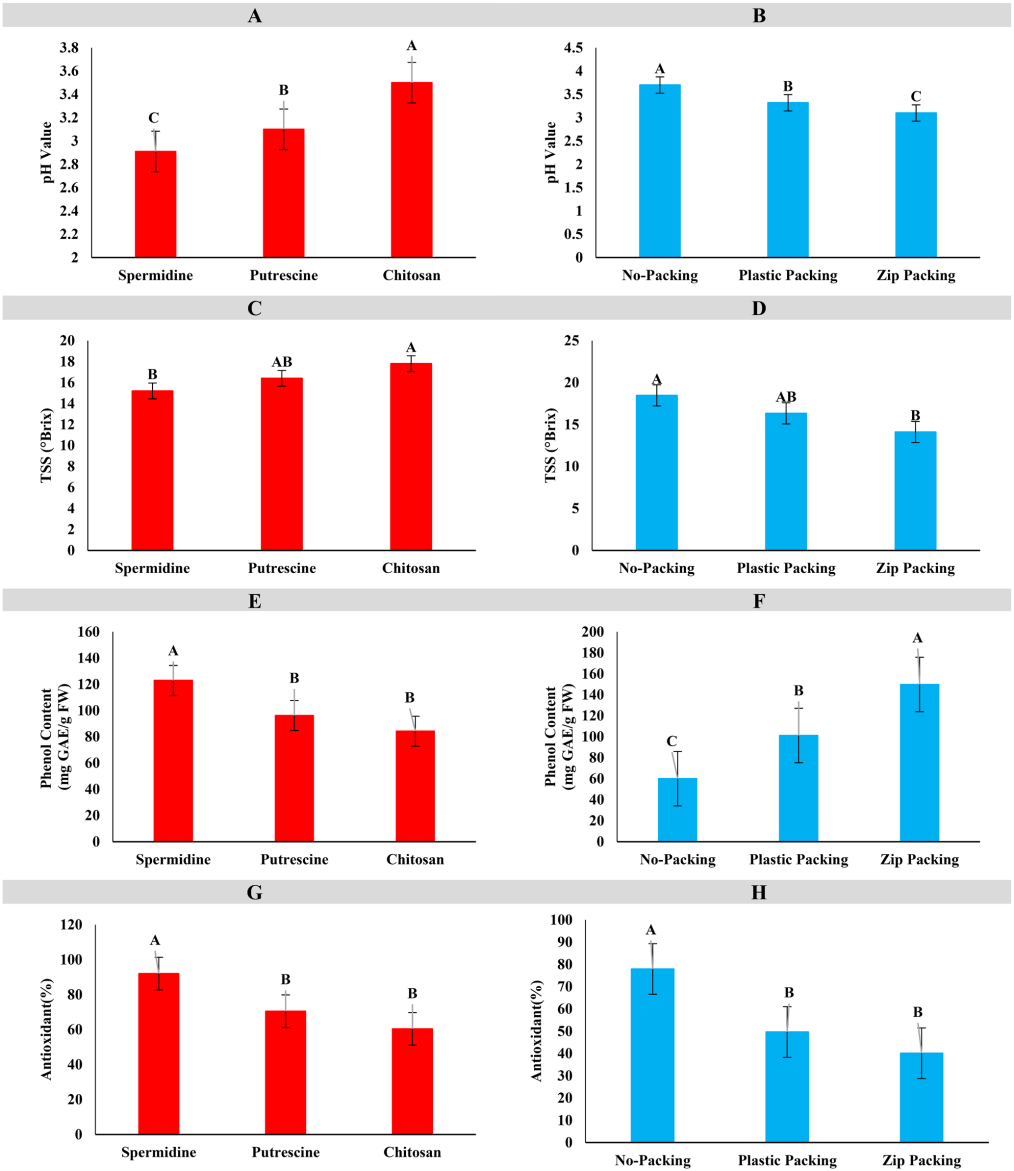
Comparison of the mean chemical components of apple fruit. In each chart, similar letters indicate no significant difference, while different letters indicate a significant difference at each level.

In Figure 5A, the effect of three coatings—spermidine, putrescine, and chitosan—on apple pH was evaluated. Chitosan exhibited the highest pH (~6.0, group A), followed by putrescine (~5.6, group B) and spermidine (~5.2, group C), indicating that the coating type significantly influences the fruit's chemical properties, with chitosan creating a more alkaline environment. Figure 5B demonstrates the impact of packaging on pH: unpackaged apples maintained the highest pH (~6.0, group A), plastic packaging resulted in moderate pH (~5.4, group B), and zip packaging caused the greatest reduction (~5.0, group C), likely due to increased acidity or enhanced gas exchange. Overall, both coating and packaging significantly affect apple pH, with chitosan coating and the absence of packaging providing optimal conditions, while spermidine and zip packaging resulted in greater pH decline. The protective effect of chitosan is attributed to the formation of a semipermeable layer that regulates gas exchange and metabolic activity, mitigating drastic chemical changes. These findings are consistent with previous studies: Çolak et al. (2025) reported that spermidine reduced pH fluctuations and preserved titratable acidity in sweet cherries; Zahedi et al. (2019) found that chitosan and spermidine treatments delayed acidity loss and stabilized pulp pH in mango; Thakur et al. (2017) observed that shrink‐wrap packaging helped maintain apple pH during storage; and Shokrallah Fam et al. (2015) demonstrated that polyamines decreased pH decline and preserved titratable acidity in plum cv. “Shablon”. Collectively, these results confirm that edible coatings such as chitosan, combined with polyamine treatments, are effective strategies for controlling pH and acidity changes, thereby reducing postharvest deterioration and extending the storage life of apples.

#### 3.2.2 Total Soluble Solids (TSS) Content of the Apple

In Figure 5C, the effect of three coatings—spermidine, putrescine, and chitosan—on apple total soluble solids (TSS) was evaluated. Chitosan exhibited the highest TSS (~18.5, group A), indicating superior preservation of sugars and soluble compounds, followed by putrescine (~17.5, group AB) and spermidine (~16.5, group B). These results suggest that coating type significantly influences internal fruit quality, with chitosan being most effective in maintaining TSS. Figure 5D illustrates the impact of packaging on TSS content: unpackaged apples showed the highest TSS (~18.5, group A), plastic packaging maintained intermediate levels (~18.3, group AB), and zip packaging caused the most significant reduction (~17.5, group B), likely due to altered moisture exchange and physiological changes. Collectively, these findings indicate that a chitosan coating combined with minimal or no packaging provides optimal conditions for preserving TSS, whereas spermidine and zip packaging result in greater declines in TSS. These observations are consistent with previous studies, including those by Hosseini et al. (2018), who reported that chitosan and putrescine treatments preserved TSS in banana cultivars, with chitosan being more effective. Similarly, Noori et al. (2024) found that spermidine maintained high TSS in “Shahroudi” grapes, while demonstrated that chitosan combined with citric acid preserved TSS in longan fruit. Moreover, Jongsri et al. (2017) (Adisak et al. 2013) observed that chitosan plus spermidine not only suppressed disease but also retained internal quality, including TSS, in “Nam Dok Mai” mango. Conversely, restrictive packaging, such as plastic or zip packs, can reduce TSS due to modified humidity, whereas lighter or no packaging better maintains soluble solids, corroborating the present results.

#### 3.2.3 Total Phenolic Content of Apple Fruit

In Figure 5E, the effect of three types of coatings—spermidine, putrescine, and chitosan—on the total phenolic content of apple fruit was evaluated. The results showed that the coatings had a statistically significant effect on this trait. The spermidine coating, with an average of about 135, was placed in statistical group A and exhibited the highest phenolic content, indicating better preservation of antioxidant compounds and bioactivity in the fruit tissue. The putrescine and chitosan coatings, with values of about 115 and 110, respectively, were assigned to group B and performed less effectively than the spermidine coating. These differences suggest that the type of coating plays a crucial role in preserving phenolic compounds, with spermidine performing best.

In Figure 5F, the effect of three types of packaging—no packaging, plastic packaging, and zip packaging—on the total phenolic content of apples was examined. Zip‐packaged samples, with a phenolic content of about 130, were placed in group A and showed the highest values. Plastic packaging, with a value of about 115, was in group B, while unpackaged samples, with a phenolic content of around 100, were in group C. These results indicate that zip packaging can better preserve phenolic compounds by reducing exposure to oxygen and moisture. In contrast, the absence of packaging led to a greater reduction in phenolic content, likely due to increased oxidative activity in the fruit.

In summary, both figures indicate that the type of coating and packaging significantly affects the maintenance of total phenolic content in apples. Spermidine coating and zip packaging provided the best conditions for preserving antioxidant compounds, whereas chitosan coating and the lack of packaging led to a greater reduction in this parameter. These findings can guide the selection of optimal methods to preserve and enhance the nutritional value and biological stability of fruits.

#### 3.2.4 The Antioxidant Content of the Apple Fruit

In Figure 5G, the effects of three coatings—spermidine, putrescine, and chitosan—on apple antioxidant content are shown. Spermidine exhibited the highest antioxidant levels (~90, group A), indicating superior preservation of bioactive compounds, followed by putrescine (~80, group B) and chitosan (~70, group B), suggesting that coating type plays a critical role in maintaining antioxidant capacity. Figure 5H illustrates the impact of packaging on antioxidant content: unpackaged apples had the highest values (~80, group A), whereas plastic (~60, group B) and zip packaging (~40, group B) showed reduced levels, likely due to altered gas and moisture conditions under more controlled packaging. Overall, these results indicate that a spermidine coating, combined with minimal or no packaging, provides optimal conditions for preserving antioxidant compounds, whereas chitosan coating and zip packaging accelerate their degradation. These findings align with prior research: Moreira et al. (2015) reported that polysaccharide‐based coatings enriched with dietary fibers and ascorbic acid preserved antioxidant properties in fresh‐cut apples, while Sharma et al. (2017) highlighted that exogenous spermidine and putrescine enhanced antioxidant enzyme activity and delayed senescence, with spermidine being particularly effective. Complementary evidence from Sharma et al. (2022) demonstrated that combining polyamine treatments with chitosan coatings synergistically maintained antioxidant activity in bell peppers. Conversely, more restrictive packaging, such as zip‐sealed containers, negatively affected antioxidant retention, consistent with the present observations.

### 3.3 Principal Component Analysis

Table 4 shows that the percentage of variance explained by principal components analysis (PCA) differs across coatings and packaging types.

**TABLE 4:** Percentage of variance explained by principal components analysis (PCA).

	PC1%	PC2%	PC3%	PC4%
Spermidine coating	99	0	0	0
Putrescine coating	96	3	0	0
Chitosan coating	75	25	0	0
No packing	84	13	2	0
Zip packing	92	8	0	0
Plastic packing	89	10	1	0

For the Spermidine coating, nearly all the variance (99%) is explained by the first component (PC1), and the other components have negligible contributions, indicating that the data variations are essentially one‐dimensional. The Putrescine coating is similar, with 96% of the variance explained by PC1 and 3% by PC2. In contrast, for the Chitosan coating, 75% of the variance is explained by PC1 and 25% by PC2, reflecting a more complex, two‐dimensional data structure.

For unpackaged samples, 84% of the variance is explained by PC1, 13% by PC2, and 2% by PC3. In contrast, for zip and plastic packaging, most of the variance is captured by PC1, with smaller contributions from PC2 and PC3. Overall, the results indicate that in most cases, the first principal component dominates, and the data variations are largely one‐dimensional. However, for specific coatings and packaging types, the second and third components also contribute significantly, suggesting a more complex structure in the dataset (Table 4).

For the Spermidine coating, the first component (PC1) has a dominant eigenvalue of 14,032.84, while the other components have much smaller values, indicating that the data variations are essentially one‐dimensional. The Putrescine coating is similar, with PC1 equal to 8343.32 and PC2 and PC3 having significantly lower values. In the Chitosan coating, PC1 is 5048.51, and PC2 is relatively high (1701.15), indicating a significant contribution from the second component and a more two‐dimensional data structure. For unpackaged samples, PC1 has an eigenvalue of 2618.03, and PC2 has an eigenvalue of 409.66, while PC3 and PC4 contribute very little. In Zip Packing, PC1 has the largest eigenvalue (16,089.54), with smaller contributions from PC2 and PC3. In Plastic Packing, PC1 dominates, and PC2 has a relative contribution of 310.41. Overall, these eigenvalues indicate that, in most cases, the first principal component accounts for the majority of the data variance. However, in some instances—such as with Chitosan coating and unpackaged samples—the second component also contributes significantly, reflecting a more complex data structure (Table 5).

**TABLE 5:** Eigenvalues of principal components (PCs) under different coating and storage conditions.

	PC1%	PC2%	PC3%	PC4%
Spermidine coating	14,032.84	62.472	25.938	3.386
Putrescine coating	8343.32	267.34	65.596	4.386
Chitosan coating	5048.5127	1701.15454	24.088	0.79950
No packing	2618.027	409.655	75.577	16.0744
Zip packing	16,089.542	1358.20813	39.661	1.654
Plastic packing	2814.65	310.408	23.2162	1.62501

**FIGURE 6:**
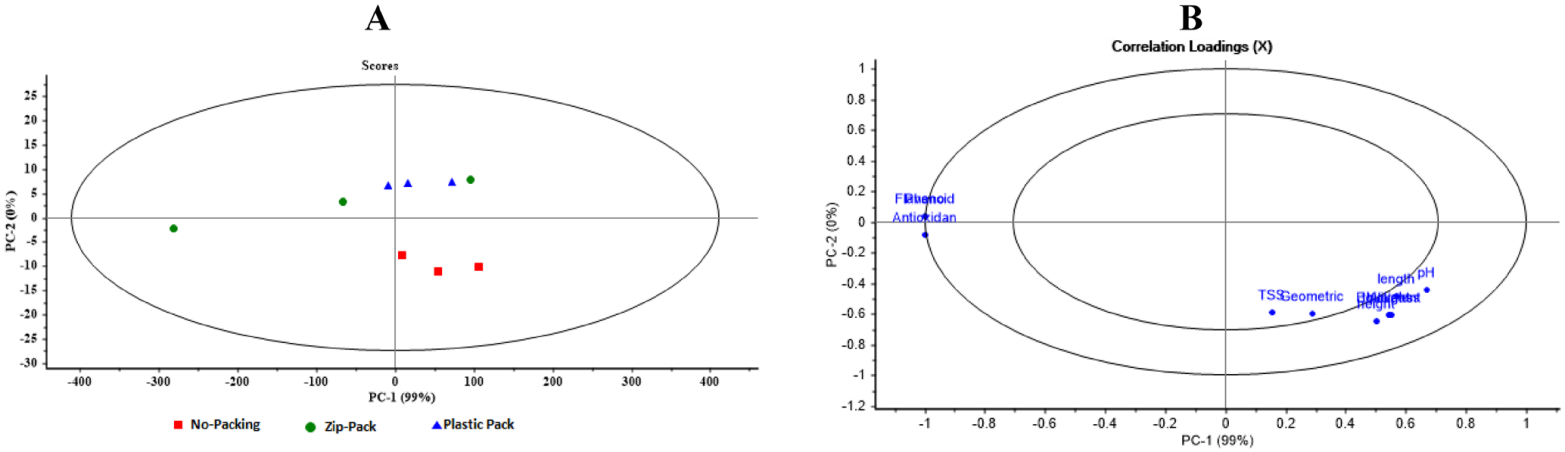
Scores and Loadings plots from principal component analysis (PCA) for distinguishing different packaging types in spermidine‐coated samples.

Figure 6 shows the Scores and Loadings plots from Principal Component Analysis (PCA) for different packaging types of Spermidine‐coated apples. In the Scores plot on the left, the samples are primarily distributed along the first principal component (PC1), which accounts for 99% of the variance. The Spermidine ‐no.pack samples, represented by blue squares, are located in the lower half of the plot, while the Spermidine ‐Zipper pack (red circles) and SperSpermidine mi‐Celloph (green triangles) cluster in the upper half and are close together. This indicates that the Zipper and Cellophane packaging share characteristics distinct from those of the unwrapped samples. In the Loadings plot on the right, variables are mapped based on their correlation with Principal Components 1 (PC1) and 2 (PC2). Most variables, such as pH, length, height, and TSS, are closely aligned with PC1 and have high loadings, indicating that PC1 explains a large portion of the data variance. Some variables, such as Antioxidant and Flavonoid, lean toward the PC2 axis, reflecting their minor contribution to the second dimension. Overall, these two plots demonstrate that PC1 plays a dominant role in differentiating the samples, while PC2 captures more minor variations. Different Spermidine packaging types are well separated based on physical and chemical characteristics.

**FIGURE 7:**
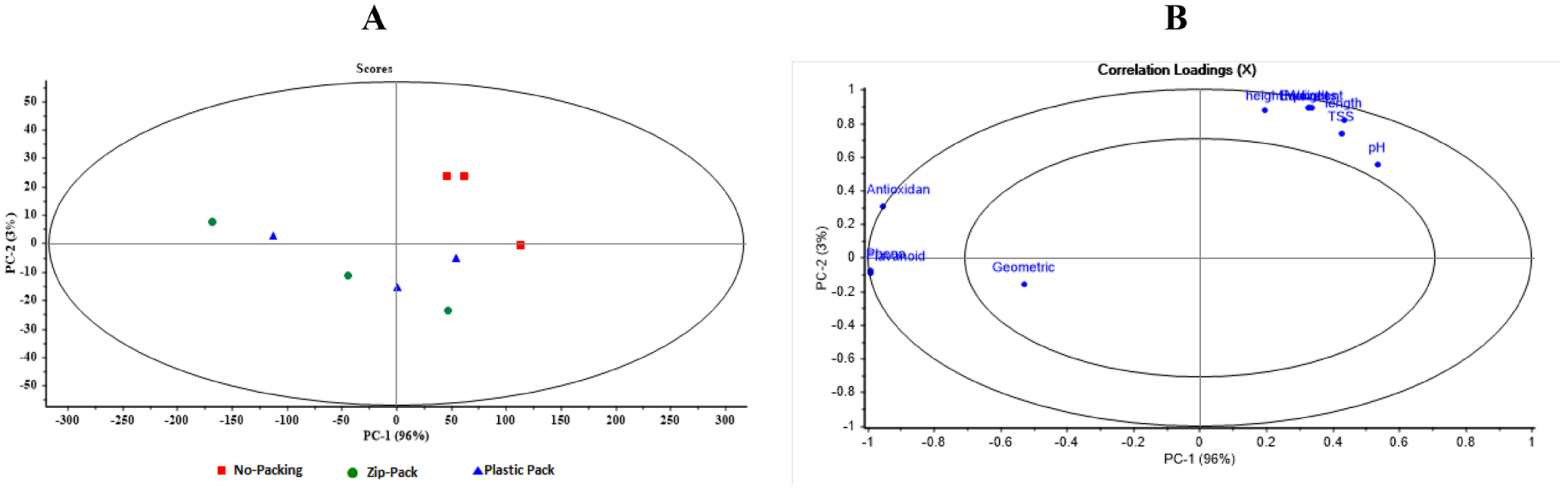
Scores and loadings plots from principal component analysis (PCA) for distinguishing different packaging types in putrescine‐coated samples.

Figure 7 presents the PCA Scores and Loadings for Putrescine‐coated apples under different packaging conditions. In the Scores plot on the left, samples are primarily distributed along PC1, which explains 99% of the variance. The unwrapped samples (blue squares) are positioned in the lower half, whereas Zipper (red circles) and Cellophane (green triangles) samples are concentrated in the upper half, indicating similarity between the Zipper and Cellophane packaging and their distinction from the unwrapped group. In the Loadings plot on the right, most variables, such as pH, length, height, and TSS, show strong loadings on PC1, indicating that PC1 explains most of the variance. Antioxidant and Flavonoid variables lean toward PC2, reflecting their minor influence on secondary separation. Overall, PC1 remains the dominant factor for distinguishing Putrescine‐coated samples, while PC2 explains minor differences.

**FIGURE 8:**
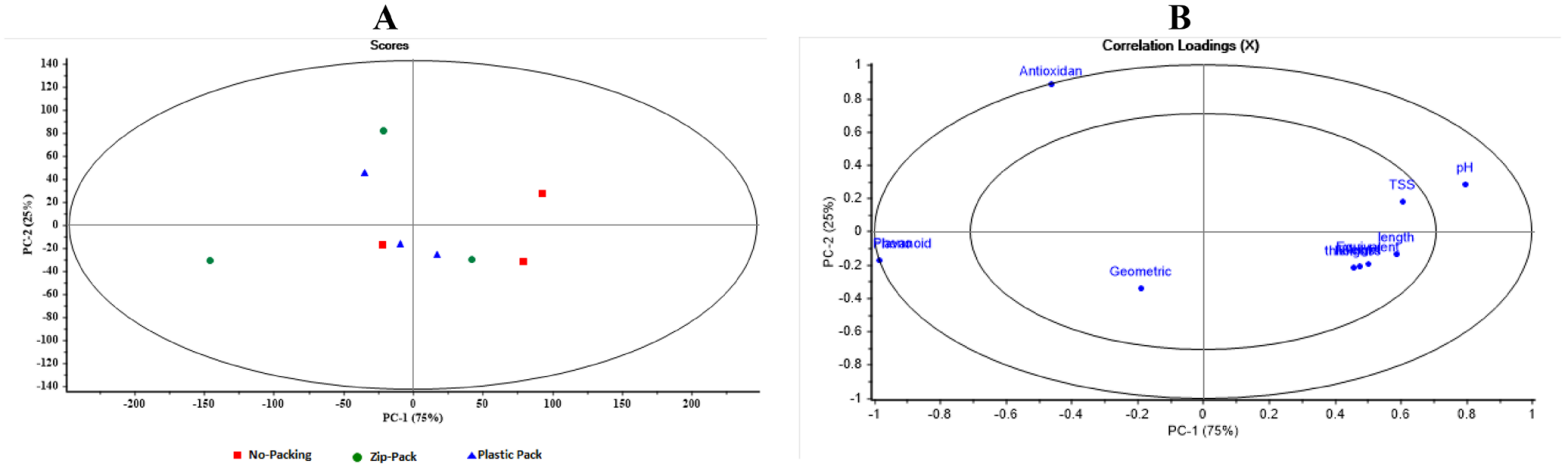
Scores and loadings plots from principal component analysis (PCA) for distinguishing different packaging types in chitosan‐coated samples.

Figure 8 displays the PCA plots for Chitosan‐coated apples with different packaging types. In the Scores plot on the left, the distribution of samples along PC1 and PC2 indicates that the blue squares (chitosan‐no.pack), red circles (chitosan‐Zipper.pack), and green triangles (chitosan‐Plastic) are relatively well‐separated. Unwrapped samples are primarily on the right, Zipper samples are on the left, and Cellophane samples are dispersed in the middle, with some overlapping points indicating similarity in certain features. In the Loadings plot on the right, variables such as pH, TSS, length, thickness, and other geometric characteristics have strong positive loadings on PC1, highlighting their role in differentiating samples. Antioxidant and Flavonoid variables show negative loadings on PC2, reflecting their minor role in secondary separation.

**FIGURE 9:**
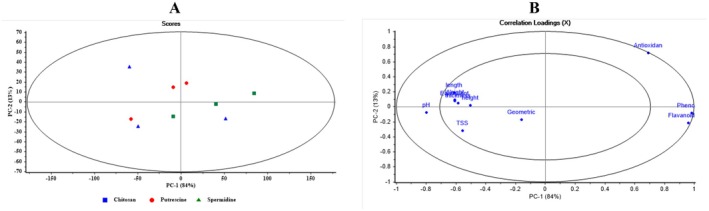
Scores and loadings plots from principal component analysis (PCA) for distinguishing different coatings in no packaging.

Figure 9 shows the PCA Scores and Loadings for different coatings in regular box packaging. In the Scores plot, samples are plotted along PC1 and PC2, with Chitosan (blue squares), Putrescine (red circles), and Spermidine (green triangles) relatively separated. PC1 explains 84% and PC2 13% of the variance. The Loadings plot shows that Antioxidant, Phenol, and Flavonoid have high positive loadings on PC1, contributing most to the differentiation of coatings. Other variables, such as pH, TSS, length, weight, and height, have moderate loadings. The combination of chemical and physical features enables PCA to effectively distinguish coatings, with PC1 playing the primary role and PC2 providing complementary separation.

**FIGURE 10:**
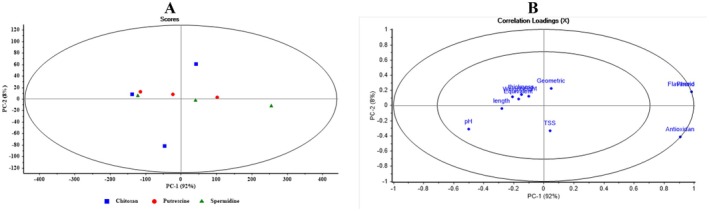
Scores and loadings plots resulting from principal component analysis (PCA) for differentiating various coatings in zip bag packaging.

Figure 10 presents PCA results for different coatings in Zipper Bag packaging. In the Scores plot, samples are distributed along PC1 and PC2 based on spectral and measured features. Spermidine samples (green triangles) are located on the right, clearly separated from Chitosan (blue squares) and Putrescine (red circles) on the left and center. PC1 explains 92% of the variance and serves as the primary factor for differentiating the data. Some minor overlap between Putrescine and Spermidine suggests a degree of similarity in certain features. In the Loadings plot, variables such as Flavonoids and Phenols have high positive loadings on PC1, and Antioxidant variables are nearby. Geometric traits, including length, width, height, and thickness, along with pH and TSS, contribute more to PC2, affecting minor distinctions. The combination of chemical and geometric features enables PCA to clearly separate Zipper Bag coatings, with PC1 serving as the primary discriminant and PC2 as a complementary axis.

**FIGURE 11:**
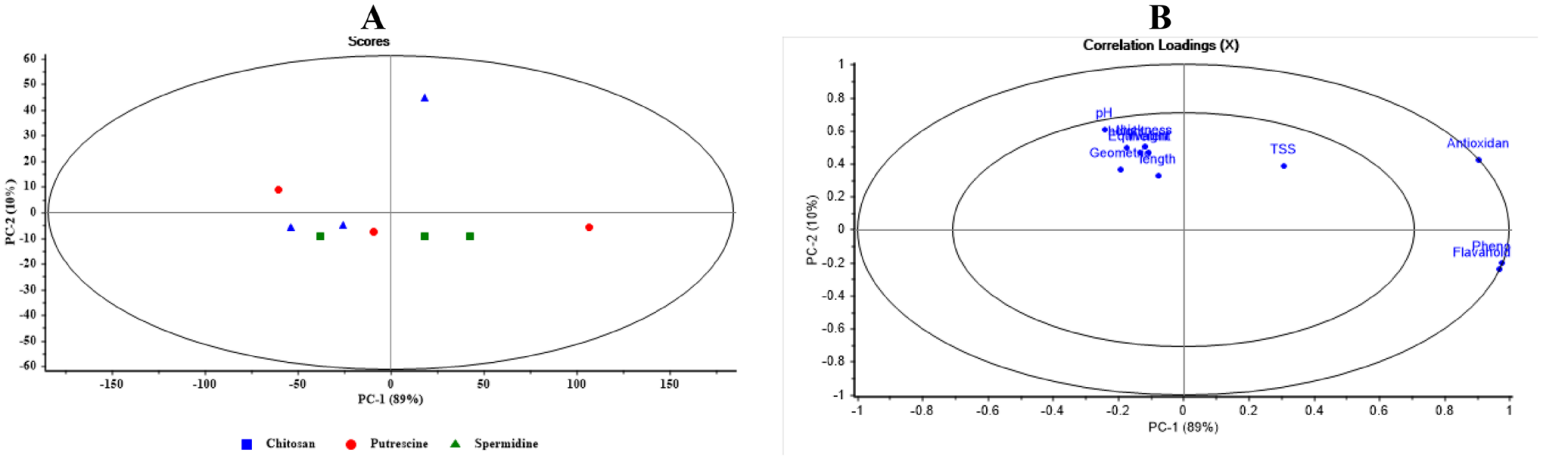
Scores and loadings plots resulting from principal component analysis (PCA) for differentiating various coatings in plastic film packaging.

Figure 11 shows PCA results for different coatings in Plastic Film packaging. In the Scores plot, Putrescine samples (red) are located on the right along PC1, clearly separated from Chitosan (blue) and Spermidine (green) on the left, indicating distinct characteristics. PC1 explains 89% of the variance, while PC2 explains 10%. In the Loadings plot, Antioxidant, Phenol, and Flavonoid have the highest positive loadings on PC1, serving as key variables for distinguishing coatings, whereas pH, TSS, and geometric features have smaller contributions. These results show that chemical antioxidants and phenolic compounds are the leading indicators for differentiating plastic film coatings, and that PCA is effective at revealing these hidden patterns.

## 4 Conclusion

Based on the precise findings from systematic measurements and statistical analyses conducted in this study, it can be concluded that the choice of coating type and packaging method significantly affects the quality and stability of red apples during storage. According to the standardized methods used to evaluate physical attributes, spermidine coating, by forming an effective protective layer, significantly minimized undesirable changes in fruit dimensions (the lowest percentage changes in length, height, and thickness) as well as weight loss (approximately 9% compared to 13%–14% for other coatings). This superiority is likely due to spermidine's role in maintaining cell membrane stability and reducing respiration rate.

On the other hand, results from the evaluation of packaging methods clearly showed that zipper bag packaging, compared to plastic packaging and unpackaged samples, created a more favorable microatmosphere, significantly preventing moisture loss and dimensional changes, and providing the best performance in preserving the fruit's physical integrity. From a chemical perspective, although packaging had a more substantial overall effect, the spermidine coating was more effective at safeguarding bioactive compounds, including phenolic and antioxidant activity. A key observation derived from the ANOVA results is the general lack of significant interaction effects, suggesting that the impacts of coating and packaging can be broadly considered and optimized independently.

However, the combined treatment of spermidine coating with zipper bag packaging was identified as the most efficient approach, integrating the advantages of both methods and achieving the most tremendous success in simultaneously maintaining the fruit's physical and chemical properties. PCA's ability to separate samples by applied treatments and to effectively identify the most influential variables (primarily physical) underscores its power in postharvest studies. Despite the comprehensive evaluation of physical and chemical attributes, this study did not include microbial or sensory analyses, which are critical components of overall fruit quality and consumer acceptance. Additionally, the storage duration was limited to 20 days, capturing only the early postharvest responses of “Red Delicious” apples. While this timeframe was sufficient to demonstrate treatment efficacy and underlying mechanisms, future research should extend the storage period (e.g., 30–60 days) and incorporate microbial safety assessments and sensory evaluation panels to provide a holistic assessment of commercial viability. Therefore, based on the applied protocols and obtained results, the practical recommendation of this study is to use a spermidine coating in combination with zipper bag packaging as a feasible, cost‐effective, and efficient solution for horticultural industries and storage facilities to extend shelf life and reduce losses of red apples.

## Author Contributions

**Mohsen Azadbakht:** writing – review and editing, writing – original draft, visualization, validation, supervision, software, resources, project administration, methodology, investigation, formal analysis, data curation, conceptualization. **Mohammad Vahedi Torshizi:** writing – review and editing, visualization, supervision, project administration, methodology, investigation, formal analysis. **Feryal Varasteh Akbarpour:** writing – review and editing, writing – original draft, visualization, validation, supervision, project administration, methodology, investigation, formal analysis, data curation. **Alireza Sabaghi Khatunabadi** and **Farimah karimzadeh viyarsagh:** writing – review and editing, writing – original draft, visualization, supervision, methodology, investigation, formal analysis.

## Consent

The authors have nothing to report.

## Conflicts of Interest

The authors declare no conflicts of interest.

## Data Availability

The data that support the findings of this study are available on request from the corresponding author. The data are not publicly available due to privacy or ethical restrictions.
